# Machine learning-based diagnosis for disseminated intravascular coagulation (DIC): Development, external validation, and comparison to scoring systems

**DOI:** 10.1371/journal.pone.0195861

**Published:** 2018-05-02

**Authors:** Jihoon G. Yoon, JoonNyung Heo, Minkyu Kim, Yu Jin Park, Min Hyuk Choi, Jaewoo Song, Kangsan Wyi, Hakbeen Kim, Olivier Duchenne, Soowon Eom, Yury Tsoy

**Affiliations:** 1 Department of Laboratory Medicine, Yonsei University College of Medicine, Seoul, Korea; 2 Department of Pharmacology, Yonsei University College of Medicine, Seoul, Korea; 3 Department of Neurology, Yonsei University College of Medicine, Seoul, Korea; 4 Solidware Inc., Seoul, Korea; Holbæk Hospital, DENMARK

## Abstract

The major challenge in the diagnosis of disseminated intravascular coagulation (DIC) comes from the lack of specific biomarkers, leading to developing composite scoring systems. DIC scores are simple and rapidly applicable. However, optimal fibrin-related markers and their cut-off values remain to be defined, requiring optimization for use. The aim of this study is to optimize the use of DIC-related parameters through machine learning (ML)-approach. Further, we evaluated whether this approach could provide a diagnostic value in DIC diagnosis. For this, 46 DIC-related parameters were investigated for both clinical findings and laboratory results. We retrospectively reviewed 656 DIC-suspected cases at an initial order for full DIC profile and labeled their evaluation results (Set 1; DIC, n = 228; non-DIC, n = 428). Several ML algorithms were tested, and an artificial neural network (ANN) model was established via independent training and testing using 32 selected parameters. This model was externally validated from a different hospital with 217 DIC-suspected cases (Set 2; DIC, n = 80; non-DIC, n = 137). The ANN model represented higher AUC values than the three scoring systems in both set 1 (ANN 0.981; ISTH 0.945; JMHW 0.943; and JAAM 0.928) and set 2 (AUC ANN 0.968; ISTH 0.946). Additionally, the relative importance of the 32 parameters was evaluated. Most parameters had contextual importance, however, their importance in ML-approach was different from the traditional scoring system. Our study demonstrates that ML could optimize the use of clinical parameters with robustness for DIC diagnosis. We believe that this approach could play a supportive role in physicians’ medical decision by integrated into electrical health record system. Further prospective validation is required to assess the clinical consequence of ML-approach and their clinical benefit.

## Introduction

Disseminated intravascular coagulation (DIC) is a life-threatening condition which arises as a secondary complication from a range of underlying conditions including sepsis, severe trauma, and advanced cancer [1]. The Scientific and Standardization Committee on DIC of the International Society on Thrombosis and Haemostasis (ISTH) define DIC as ‘an acquired syndrome characterized by the intravascular activation of coagulation with a loss of localization arising from different causes.’[2] Despite this definition highlighting DIC’s key features, the major challenge in the diagnosis of disseminated intravascular coagulation (DIC) comes from the lack of a single potent marker for DIC, leading to developing composite scoring systems, derived from underlying conditions and laboratory results [2–4].

The diagnostic criteria, widely used as a gold standard, is the ISTH criteria which consist of platelet (PLT) count, prothrombin time (PT), fibrinogen, and fibrin-related markers (e.g. D-dimer or fibrin degradation products; FDP) [2]. Although the ISTH criteria have been validated by various studies and the performance was shown to be satisfactory, several issues remain [5–8]. Particularly, determination of the optimal fibrin-related markers and individual laboratory cut-off values for moderate to strong increase have not yet been clearly defined [9–12]. Furthermore, the ISTH criteria’s sensitivity is regarded by some to be lacking when compared to other scoring systems [13]. Two other well-established scoring systems are the Japanese Ministry of Health and Welfare’s criteria (JMHW criteria) and the Japanese Association for Acute Medicine’s criteria (JAAM criteria; Table 1) [4–14]. Those criteria have respective advantages and limitations depending on the underlying conditions, and numbers of refinements have been made [10, 13, 15].

**Table 1:** Diagnostic scoring systems for disseminated intravascular coagulation (DIC) used in this study.

DIC criteria	ISTH	JMHW	JAAM
Underlying condition	Essential	1 p	Essential
Clinical symptoms	NA	Bleeding: 1 p	SIRS score ≥ 3: 1 p
		Organ failure: 1 p	
Prothrombin time (PT)	Prolonged PT (sec)	PT ratio	PT ratio
	3 <–≤ 6: 1 p	1.25 ≤–< 1.67: 1 p	≥ 1.2: 1 p
	> 6: 2 p	≥ 1.67: 2 p	
Platelet count (×10^3^/μL)	50 ≤–< 100: 1 p	80 <–≤ 120: 1 p	80 ≤–≤ 120 or > 30% reduction/24h: 1 p
	< 50: 2 p	50 <–≤ 80: 2 p	< 80 or > 50% reduction/24h: 3 p
		≤ 50: 3 p	
Fibrin-related marker	FDP, D-dimer, SF	FDP (μg /mL)	FDP (μg /mL)
	Moderate increase: 2 p	10 ≤–< 20: 1 p	10 ≤–< 25: 1 p
	Strong increase: 3 p	20 ≤–< 40: 2 p	≥ 25: 3 p
		≥ 40: 3 p	
Fibrinogen (mg/dL)	< 100: 1 p	100 <–≤ 150: 1 p	NA
		≤ 100: 2 p	
Score range	0–8 p	0–13 p	0–8 p
DIC diagnosis	≥ 5 p	≥ 7 p	≥ 4 p

Abbreviations: ISTH, the International Society on Thrombosis and Haemostasis; JMHW, the Japanese Ministry of Health and Welfare; JAAM, the Japanese Association for Acute Medicine; NA, Not applicable; SF, soluble fibrin; p, point

Artificial intelligence (AI)—where computers mimic human intelligence through machine learning algorithms—has drawn media attention, and ubiquitous application of AI has grown in momentum across various fields. Similar trials have shown up in the medical field, particularly using clinical data and medical images [16–19]. Artificial neural network (ANN) resembles human neuronal connections by building a multi-layered network and can be trained to functionalize or categorize complex patterns [20, 21]. There are two remarkable characteristics of this machine learning (ML)-approach: 1) non-linear pattern recognition and 2) improvement by learning. These features are not only ideal for considering various clinical conditions, but also for giving standardized results with wide extensibility. ANNs have demonstrated positive medical application in areas such as diagnosis of myocardial infarction, cancer, and diabetic retinopathy [22–24]. In this study, we demonstrated ML-approaches for DIC diagnosis and established an optimized ANN model which integrates both the clinical findings and the laboratory results.

## Materials and methods

### Patients

This study was approved by the institutional review board and the ethics committee of Yonsei University Health System (Seoul, Korea; IRB 4-2016-0698). The current study used medical records and participating centers have waived by completing the questionnaires. All data was treated confidentially with anonymized numbers. Patients with full DIC profile were defined as cases with all laboratory results including complete blood count (CBC) with differential counts, global coagulation tests (PT, PT % activity, international normalized ratio [INR], activated partial thromboplastin time [aPTT], and thrombin time), fibrinogen, D-dimer, FDP, and anti-thrombin III (AT III) having been ordered on the same day (this order set defined as ‘DIC profile’).

**Fig 1:**
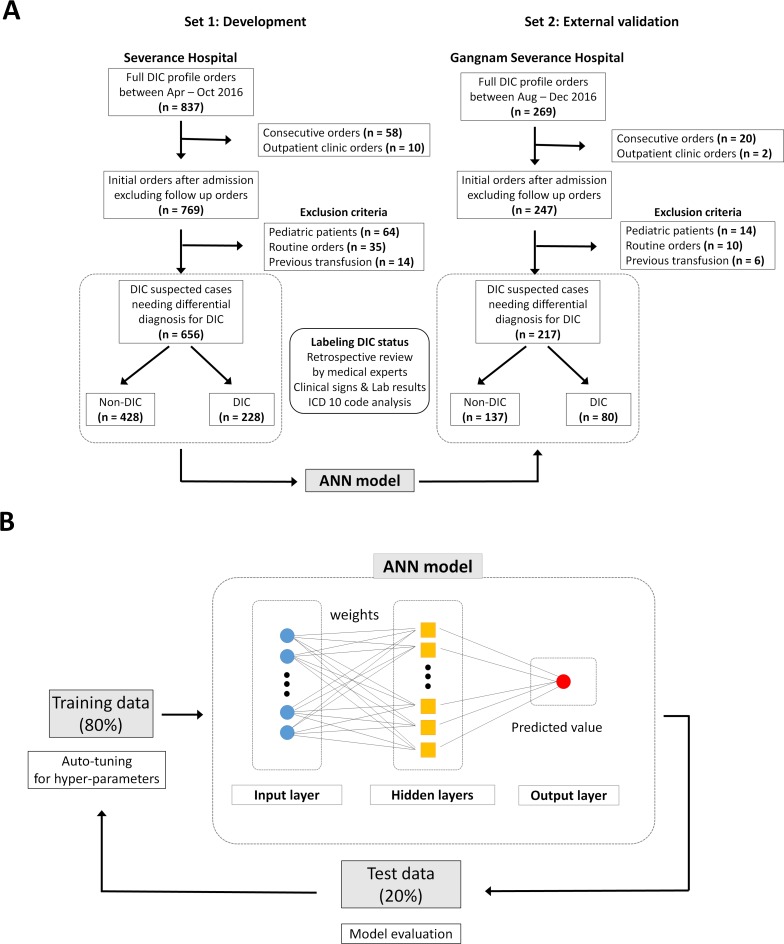
Schematic representation of patient enrollment and development of the artificial neural network (ANN) model. (A) Full DIC profile was defined as all laboratory results including complete blood count with differential counts, global coagulation tests (PT, PT % activity, international normalized ratio [INR], activated partial thromboplastin time [aPTT], and thrombin time), fibrinogen, D-dimer, FDP, and anti-thrombin III. The external validation hospital used different DIC profile: protein C was included instead of FDP, and RUO parameters were not provided. (B) ANN model for DIC diagnosis. In the training phase, the development set (n = 656) was randomly split into training and test sets in 80:20 ratio and hyper-parameters were determined for an optimal modeling. All layers have 32 nodes with an input-layer and two-hidden layers. The relative importance of input features was calculated based on the ‘Connection Weight’ approach, after the ANN model was established.

Eligible cases had full DIC profile orders (n = 837) between April and October 2016 at a tertiary hospital (Severance Hospital, Seoul, Korea; Fig 1A). After excluding consecutive orders from the identical patients and outpatient clinic orders, patients with initial full DIC profile after admission (n = 769) were enrolled. Cases from pediatric patients, routine orders at admission, long-term hospitalized patients, or previous transfusion therapy were excluded (n = 113). Finally, the development set (set 1; n = 656) remained with DIC suspected cases requiring an evaluation of DIC. Because the cases were enrolled at initial evaluation point, no cases were previously treated with transfusion therapy (plasma product or cryoprecipitate) or AT III. A similar approach was used for the external validation set (set 2; n = 217) derived from data obtained from another tertiary hospital (Gangnam Severance Hospital, Seoul, Korea). Demographics and clinical characteristics of the patients are represented in Table 2. There were no cases having heparin-induced thrombocytopenia, thrombotic microangiopathy (TMA), or antiphospholipid syndrome.

**Table 2:** Patient demographics and clinical characteristics.

Numbers of cases (%)	Development set (set 1; n = 656)			External validation set (set 2; n = 217)		
	DIC (n = 228)	Non-DIC (n = 428)	*P* value	DIC (n = 80)	Non-DIC (n = 137)	*P* value
Age, years	60.8 ± 16.6	58.1 ± 17.5	.053	62.7 ± 15.9	64.2 ± 16.5	.228
Gender, male	119 (52.2)	225 (52.6)	.992	49 (61.3)	71 (51.8)	.530
Currently in ICU	155 (68.0)	171 (40.0)	< .001	59 (73.8)	59 (43.1)	< .001
APACHE II score ± SD *	30.2 ± 10.7	24.3 ± 9.8	< .001	25.3 ± 9.7	16.2 ± 6.7	< .001
Mortality (28-days)	146 (64.0)	77 (18.0)	< .001	53 (66.2)	12 (8.8)	< .001
On anticoagulation therapy	2 (0.9)	32 (7.5)	.001	2 (2.5)	5 (3.6)	.949
Thrombosis	12 (5.3)	46 (10.7)	.027	4 (5.0)	9 (6.6)	.862
Bleeding	60 (26.3)	85 (19.9)	.072	22 (27.5)	12 (8.8)	.001
Organ failure	116 (50.9)	58 (13.6)	< .001	39 (48.8)	15 (10.9)	< .001
SIRS score ≥ 3	176 (77.2)	146 (34.1)	< .001	66 (82.5)	53 (38.7)	< .001
Associated conditions with DIC						
Sepsis/Infection	161 (70.6)	140 (32.7)	< .001	58 (72.5)	47 (34.3)	< .001
Tissue damage	28 (12.3)	51 (11.9)	.991	23 (28.7)	33 (24.1)	.551
Post major surgery	34 (14.9)	133 (31.1)	< .001	13 (16.2)	46 (33.6)	.009
Hematologic malignancy	26 (11.4)	35 (8.2)	.225	7 (8.8)	5 (3.6)	.201
Solid cancer	87 (38.2)	82 (19.2)	< .001	20 (25.0)	17 (12.4)	.028
Hepatic failure	34 (14.9)	17 (4.0)	< .001	13 (16.2)	4 (2.9)	.001
Obstetric complications	4 (1.8)	21 (4.9)	.073	1 (1.2)	3 (2.2)	> .999
Vascular abnormalities	4 (1.8)	18 (4.2)	.152	11 (13.8)	31 (22.6)	.156
Immunologic insult	4 (1.8)	22 (5.1)	.057	9 (11.2)	5 (3.6)	.056

* The Acute Physiology and Chronic Health Evaluation (APACHE) II score was calculated only if a patient was admitted to ICU.

### Data collection and labeling DIC status

**Fig 2:**
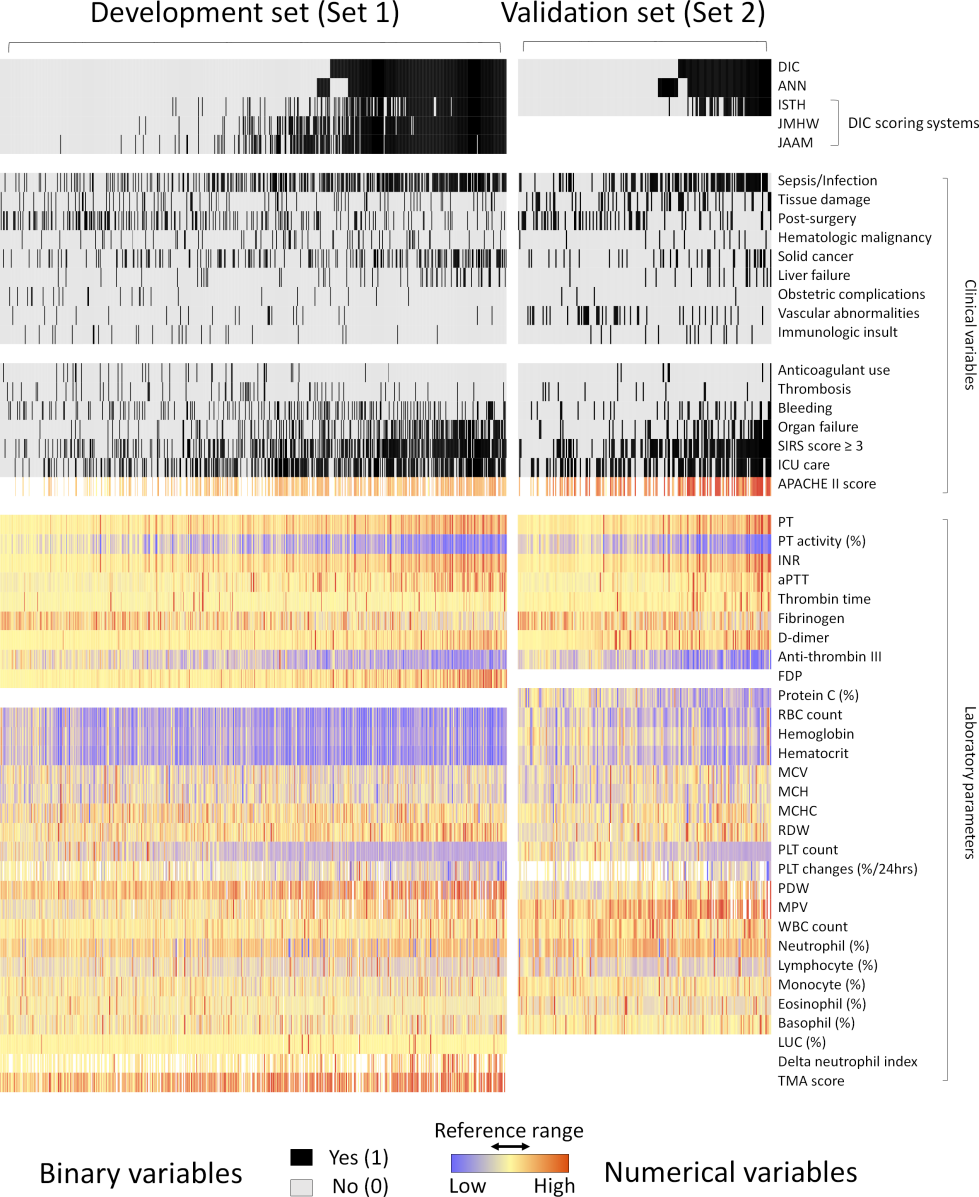
Heat map presentation of the datasets used in this study. The x-axis denotes individual cases and the y-axis corresponds to the clinical variables. Each cell shows values of variables for each case. All cases are sorted horizontally by the labeled DIC status and predicted ANN model values. Rows 2–5 (ANN model, ISTH, JMHW, and JAAM criteria) show predictions of different DIC diagnostic classifiers based on the cut-off values (0.501 for ANN) or points (Table 1).

We retrospectively followed the timeline of physicians’ diagnostic process. Therefore, the presence of clinical signs, symptoms, underlying DIC-related conditions and the full set of laboratory results was obtained at the same day of DIC profile (Tables 2 and 3, Text A in S1 File) *(9)*. CBC was obtained from K_2_-EDTA tube using automated hematology analyzers (ADVIA 2120i; Siemens Healthcare Diagnostics, IL, USA) which provide commonly reported clinical parameters and additional research use only (RUO) parameters, such as large unstained cells (LUC; %), delta neutrophil index (DNI), and TMA score [25–27]. Global coagulation tests and fibrin-related markers were performed using ACL-TOP 750 analyzer (Instrumentation Laboratory, Bedford, MA, USA), with the samples collected in 3.2% sodium citrate tubes. Noticeably, the external validation hospital used different automated hematologic analyzers (XN-9000 and CS-5100 system; Sysmex, Kobe, Japan) and had different DIC profile: protein C was included instead of FDP, and RUO parameters were not provided. In this reason, four parameters were excluded in set 2 (Fig 2). To curate the DIC status (non-DIC: 0, DIC: 1), patient’s medical record, clinical manifestation, and laboratory results were retrospectively reviewed by medical experts. Each case was carefully reviewed by two experts individually and the patient’s evaluation result which occurred within a week was assigned comprehensively depending on the laboratory data change, clinical manifestation, clinical intervention, and final diagnosis. If a discrepancy occurred, the case was reviewed by another third expert and labeled after a consensus was reached.

**Table 3:** Laboratory results for full DIC profile parameters.

Parameters, mean (SD)	Development set (set 1; n = 656)			External validation set (set 2; n = 217)		
	DIC (n = 228)	Non-DIC (n = 428)	*P* value	DIC (n = 80)	Non-DIC (n = 137)	*P* value
Global coagulation tests with DIC profile						
Prothrombin time (PT; sec)	21.9 (10.3)	15.1 (6.5)	< .001	20.6 (7.4)	14.6 (3.9)	< .001
PT activity (%)	50.4 (18.8)	75.8 (20.9)	< .001	50.4 (17.9)	77.7 (18.7)	< .001
INR	1.92 (0.90)	1.32 (0.56)	< .001	1.72 (0.65)	1.21 (0.34)	< .001
aPTT (sec)	60.3 (36.5)	39.1 (18.8)	< .001	52.2 (35.6)	31.3 (11.0)	< .001
Thrombin time (sec)	21.5 (17.0)	20.0 (18.2)	.297	24.9 (13.9)	19.3 (3.0)	< .001
Fibrinogen (mg/dL)	304 (189)	415 (178)	< .001	326 (212)	470 (202)	< .001
D-dimer (μg/mL)^a^	8.23 (12.95)	2.10 (4.55)	< .001	16.96 (18.86)	7.25 (12.16)	< .001
Anti-thrombin III activity (%)	53.8 (20.0)	81.0 (24.6)	< .001	50.5 (19.9)	79.8 (18.8)	< .001
Fibrin degradation product (μg /mL)	59.4 (62.3)	18.0 (24.6)	< .001	NA^e^	NA	NA
Protein C activity (%)	NA	NA	NA	40.7 (19.5)	81.9 (31.4)	< .001
Complete blood count and related parameters						
RBC count (×10^6^/μL)	2.97 (0.65)	3.25 (0.69)	< .001	3.20 (0.89)	3.56 (0.74)	.002
Hemoglobin (g/dL)	9.2 (2.0)	10.0 (2.0)	< .001	9.9 (2.7)	10.9 (2.0)	.003
Hematocrit (%)	27.5 (5.7)	29.8 (5.9)	< .001	29.8 (8.1)	33.0 (6.4)	.002
MCV (fL)	93.0 (6.8)	92.3 (5.9)	.150	93.5 (6.3)	92.9 (5.7)	.477
MCH (pg)	31.1 (2.3)	30.8 (2.1)	.038	31.0 (2.4)	30.7 (2.4)	.412
MCHC (g/dL)^b^	33.4 [32.4, 34.4]	33.4 [32.5, 34.2]	.433	33.0 [32.1, 34.1]	33.0 [32.4, 33.6]	.334
RDW (%)	16.5 (2.7)	15.4 (2.4)	< .001	15.5 (2.8)	14.1 (2.0)	< .001
PLT count (×10^3^/μL)	57 (56)	168 (129)	< .001	77 (78)	211 (133)	< .001
PLT changes (%/24hrs)^c^	-0.36 (0.60)	-0.15 (0.33)	< .001	-0.40 (0.52)	-0.08 (0.32)	< .001
PDW (%, fL)^d^	61.9 (17.2)	56.4 (10.6)	< .001	13.7 (3.7)	11.5 (2.2)	< .001
MPV (fL)	10.45 (1.99)	9.15 (1.49)	< .001	11.36 (1.99)	10.33 (1.49)	< .001
WBC count (×10^3^/μL)	12.75 (17.60)	10.60 (9.61)	.044	11.75 (17.60)	13.40 (9.61)	.218
Differential count (%)						
Neutrophil	77.7 (24.0)	75.5 (21.0)	.226	79.9 (15.4)	78.0 (17.3)	.404
Lymphocyte	13.2 (19.3)	14.4 (16.3)	.423	12.5 (15.4)	13.5 (12.7)	.626
Monocyte	4.8 (3.6)	5.5 (4.5)	.063	6.0 (4.4)	7.0 (6.9)	.250
Eosinophil	0.8 (1.5)	1.8 (3.8)	< .001	1.1 (1.8)	1.3 (1.9)	.566
Basophil	0.2 (0.3)	0.2 (0.2)	.845	0.4 (0.7)	0.3 (0.3)	.114
Large unstained cell (LUC)	3.2 (13.0)	2.2 (8.6)	.277	NA	NA	NA
Delta neutrophil index (%)	13.5 (16.2)	5.0 (9.2)	< .001	NA	NA	NA
Thrombotic microangiopathy score	2.7 (1.5)	2.0 (1.4)	< .001	NA	NA	NA

^a^ measured in data display unit (DDU; set 1) or fibrinogen-equivalent unit (FEU; set 2). See Table A in S1 File.

^b^ Non-parametric distribution, median [25, 75 percentiles].

^c^ The percent change in PLT count within 24 hours was calculated only if previous PLT count was available.

^d^ The report units are different for PDW value between set 1 (%) and set 2 (fL).

^e^ NA, not applicable

### Model development

Using the collected datasets, we tested several ML algorithms including logistic regression, linear regression, ridge regression, random forest, gradient boosting machine, deep learning, and ANN with DaVinci Labs (Solidware Inc., Seoul, Korea) which support an AI-based data analysis; the performance of ANN model was the best among the seven algorithms. In the training phase, the development set was randomly split into training and test sets in 80:20 ratio. Next, auto-tuning for hyper-parameters (e.g. number of hidden layers, epochs) with respect to the performance of the model on the test set was conducted. After several iterations of the auto-tuning and training processes, an ANN model (2 hidden layers, 10 epochs) to evaluate DIC status was established (Fig 1B). Additionally, we calculated the relative importance of the input variables with ‘Connection Weight’ approach [28]. Briefly, the importance of variables is proportional to the sum of absolute values of products between weights of connections, by which this variable is propagated. We also conducted an external validation of the ANN model using set 2. As set 2 was missing four variables, the ANN model was re-trained without the four variables and evaluated.

### Analysis

Performance of the ANN model was compared to the three scoring systems (ISTH, JMHW, and JAAM; Table 1). Sensitivity, specificity, positive and negative predictive value, and area under curve (AUC) values were calculated following each criterion. D-dimer was used as the fibrin-related marker for the ISTH criteria as both set 1 and set 2 had this parameter (cut-off values for the moderate to the strong increase were based on 25% and 75% quartiles of all patients in each hospital, respectively.) [9]. JMHW and JAAM scores could not be evaluated in set 2 as FDP was lacked in the DIC profile of set 2.

Statistics software R version 3.4.3 was used for data analysis. Datasets were visualized using ‘*ComplexHeatmap*’ package [29]. Performance evaluation was achieved via receiver operating characteristic (ROC) curve analysis, and calculation of AUC using the ‘*pROC*’ package [30]. The cut-off value (0.501) for the ANN model was determined by the ‘*OptimalCutpoints*’ package using the Youden method [31]. Statistical analyses were performed by Student’s *t*-test for parametric data and Mann-Whitney U test for non-parametric data. *P* values below 0.05 were considered as statistically significant.

## Results

### Patient characteristics

We conducted a retrospective cross-sectional study of DIC-suspected patients at initial evaluation with full DIC profiles. All available cases with full DIC profiles were reviewed in two different hospitals. After excluding cases from consecutive orders, pediatric patients, routine orders at admission or from long-term hospitalized patients, and patients with previous transfusion therapy, the development dataset was constructed from 656 patients with initial full DIC profiles. Among the 656 patients admitted to either general ward (n = 330) or intensive care unit (ICU; n = 326) in Set 1, 228 (34.8%) and 428 (65.2%) patients were labeled as DIC and non-DIC status, respectively (Table 2). Univariate analysis showed no differences in age or gender between the two groups (DIC vs. non-DIC; in the parentheses). However, the proportion in ICU (68.0 vs. 40.0%, *P* < .001), Acute Physiology and Chronic Health Evaluation (APACHE) II scores (30.2 vs. 24.3, *P* < .001) which were only calculated for ICU patients [32], and 28-days mortalities (64.0 vs. 18.0%, *P* < .001) were higher in DIC group. Moreover, the proportion of patients showing organ failure (50.9 vs. 13.6%, *P* < .001) and systemic inflammatory response syndrome (SIRS; 77.2 vs. 34.1%, *P* < .001) was higher in the DIC group. Additionally, bleeding was more common in the DIC group (26.3 vs. 19.9%) although the difference was only significant at *P* = .072. Above clinical conditions showed similar results in Set 2.

We also investigated the DIC-related conditions, and sepsis/infection was the most common condition (70.6%) followed by solid cancer (38.2%). Sepsis/Infection (70.6 vs. 32.7%, *P* < .001), solid cancer (38.2 vs. 19.2%, *P* < .001), and hepatic failure (14.9 vs 4.0%, *P* < .001) were the underlying conditions positively correlated with the DIC group. While post major surgery status (14.9 vs 31.1%, *P* < .001) tended to be more prevalent in the non-DIC group, we believe that this was resulted by physicians’ inclination ordering DIC profile after major surgery. Other associated conditions such as tissue damage, hematologic malignancy, obstetric complications, vascular abnormalities, and toxic or immunologic insult showed no significant result between the two groups, although the number of such cases was relatively small.

Most laboratory results showed a difference between the DIC and non-DIC groups. Global coagulation parameters, except thrombin time, showed different results (*P* < .001; Table 3). CBC components also presented different results, except for two RBC indices and some differential counts. The relative lower levels of RBC count and hemoglobin in the DIC group may be caused by the higher proportion of bleeding patients than the non-DIC group. We visualized the two datasets with the heat map (Fig 2) which enabled us to look over the landscapes of the data distributions. Sepsis/infection, SIRS, and ICU admission were more commonly observed in the DIC group. PT and PLT count showed reverse predisposition, as expected. The general patterns represented in the heat map confirmed the similar composition of the two data sets, while vascular abnormalities were more common in the validation set due to the vascular surgery center located at this hospital. Missing values were presented as blanks, and seven variables contained missing values. APACHE II scores were only calculated if a patient was admitted to ICU. PLT changes (%/24hr) were only available for patients with previous PLT count result. WBC differential counts, PLT distribution width (PDW), Mean PLT volume (MPV), and RUO parameters were not reported from hematologic analyzers in cases of severe thrombocytopenia or leukopenia.

### Established model and variable importance

We first tested the ANN model with 46 investigated variables and gradually excluded negligible variables. The laboratory parameters with trivial impacts on the performance were excluded: mean corpuscular volume (MCV), mean corpuscular hemoglobin (MCH), mean corpuscular hemoglobin concentration (MCHC) and WBC counts. Because of the small number of the cases with trivial effects, the following clinical variables were also excluded: anticoagulant use, bleeding, thrombosis, hematologic malignancy, immunologic insult, hepatic failure, obstetric complication, tissue damage, and vascular abnormalities. Consequently, 32 representative variables were used in the developed ANN model including clinical signs and symptoms, underlying conditions, and laboratory parameters.

**Fig 3:**
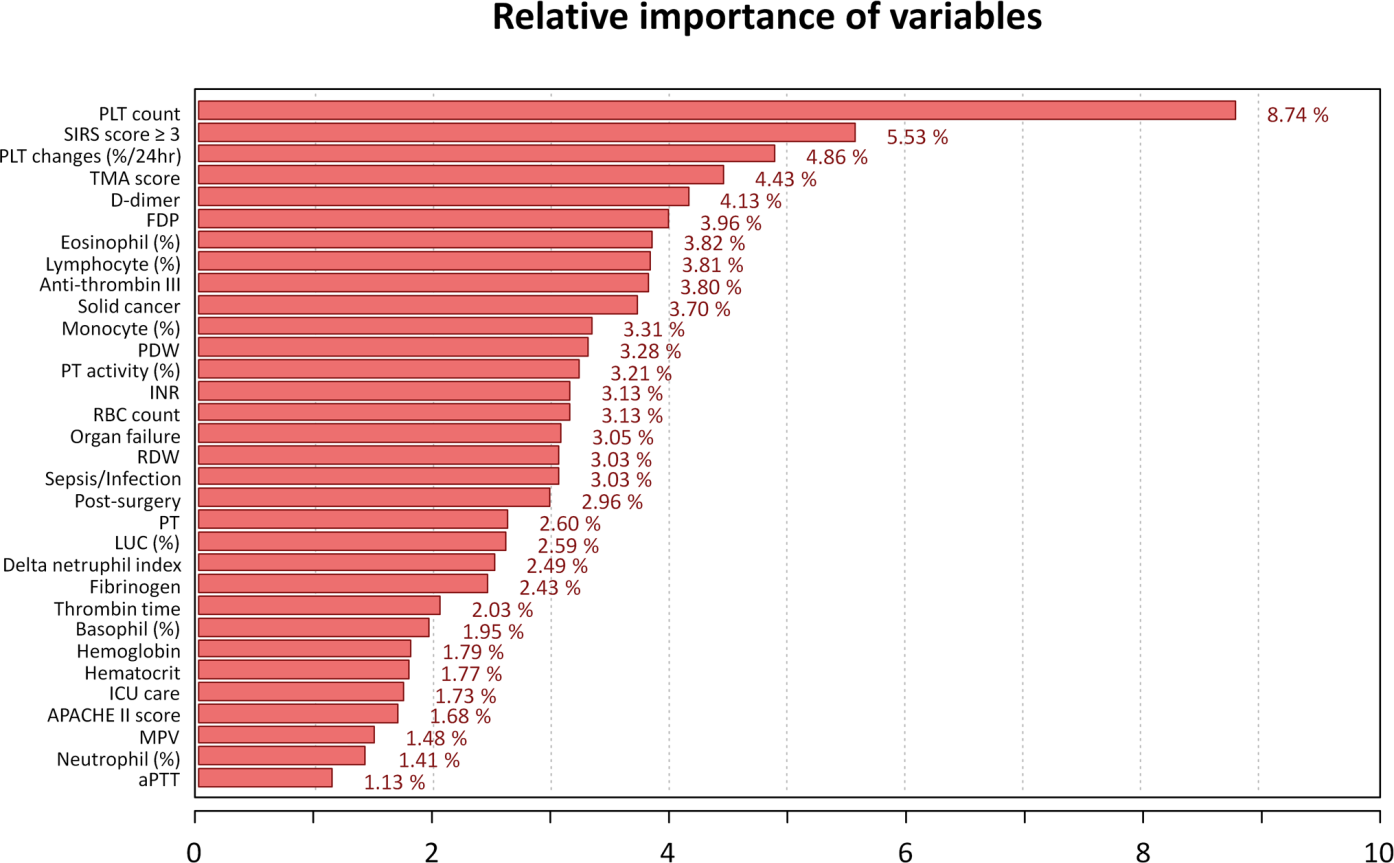
Relative importance of clinical and laboratory variables in the ANN model. In the developed ANN model, 32 variables are used and their relative importance is calculated based on the weight value, reflecting connectivity of neurons, using ‘Connection Weight’ approach to provide explanatory insights for each variable. (Total sum: 100%, average importance: 3.13%).

To provide an interpretable model for each clinical variable, we calculated the relative importance. It is noteworthy that statistical significance does not guarantee variable importance level in ANN, and vice versa. Recently, the ‘Connection Weight’ approach was reported to be an efficient method of identifying variable importance in ANN model [33]. Fig 3 shows the calculated importance using this approach in 32 clinical variables. Most parameters such as PLT count (8.74%), PLT changes (4.86%), D-dimer (4.13%), and FDP (3.96%) had contextual importance in accordance with DIC features, whereas fibrinogen level (2.43%) showed relatively low importance. PLT count and PLT changes ranked as the first and third important variable and these results were expectable owing to the evident statistical differences between the DIC and non-DIC groups (57 vs. 168 ×10^3^/μL, *P* < .001; -0.36 vs. -0.15, *P* < .001). To minimize inter-laboratory variations, three PT parameters (sec, INR, percent activity) were separately used in the model, because INR and PT % activity is a standardized value using normal pooled plasma. Although separately evaluated, PT parameters occupied a total of 8.94% of the entire importance level and also played a significant role. Interestingly, the importance of previously overlooked parameters was not negligible in the ANN model including PDW (variations in PLT size and shape), red cell distribution width (RDW; variations in RBC size and shape), and RUO parameters. Most parameters presented a contextual importance, however, their importance in the ML-approach was different from the traditional approach.

### Performance

**Fig 4:**
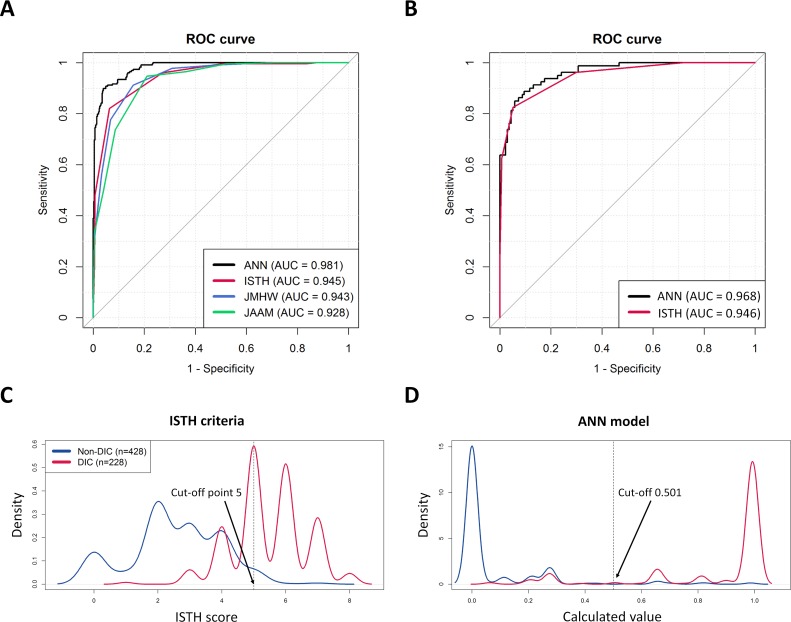
Diagnostic performance of ANN model and scoring systems with receiver operating characteristic curve analysis and density plot. (A) Training (Set 1): ANN model shows the best performance among the four diagnostic classifiers. The area under curve (AUC) values: ANN (0.981), ISTH (0.945), JMHW (0.943), and JAAM (0.928). (B) External validation (Set 2): four variables were unavailable owing to the different hematologic analyzers, therefore the AUC value was compromised compared to the development set in the ANN model; ANN (0.968), ISTH (0.946). (C, D) Density plots of two represented diagnostic classifiers (ANN model, ISTH criteria) shows that the ANN model far obviously differentiates two groups (DIC and non-DIC). The cut-off value for the ANN model is determined at 0.501.

The performance of four methods was compared in terms of AUC values, sensitivity, specificity, and predictive values (Table 4). Among the four methods, the ANN model showed the best AUC value with *P* < .001 while the three DIC criteria presented no differences (Fig 4A). The AUC (95% confidence interval; CI) of the ANN model was 0.981 (0.973–0.989) and the three DIC criteria had AUC of 0.945 (0.929–0.962) for the ISTH, 0.943 (0.927–0.959) for the JMHW, and 0.928 (0.909–0.946) for the JAAM, respectively. The optimal cut-off value by Youden index for the ANN model was 0.501 with 89.9% (85.2–93.5) and 96.0% (93.7–97.7) of the sensitivity and specificity (95% CI), respectively. Additionally, the sensitivity and specificity of the three DIC criteria were 82.0% (76.4–86.8), 93.7% (91.0–95.8) for the ISTH, 91.2% (86.8–94.6), 84.3% (80.6–87.7) for the JMHW, and 94.7% (91.0–97.3), 79.0% (74.8–82.7) for the JAAM, respectively. All methods showed relatively lower performance than the previous prospective study in the ICU setting using the ISTH criteria (sensitivity 91%, specificity 97%) [5]. The difference may be attributed to the study design, the patient composition and ward setting, the hematologic analyzer, and/or variance in the expert opinion. Nevertheless, the ISTH criteria showed relatively low sensitivity and high specificity, while the JMHW showed relatively high sensitivity and low specificity, as reported [9]. Furthermore, we reviewed the performance in the external validation set (n = 217). The ANN model was re-trained without the four variables (FDP and RUO parameters) which were included in set 1, and the AUC value of this model without the four variables was 0.975 (0.966–0.984). Using this model, we tested set 2, and the AUCs were 0.968 (0.945–0.986) for the ANN model and 0.946 (0.916–0.976) for the ISTH (Fig 4B, Table 4). Both models showed slightly compromised results, while the ISTH remained with constant AUC with slightly skewed performance–low sensitivity and high specificity. The compromised AUC portion in the ANN model may primarily come from the different hospital setting, the different reference intervals, and/or the unstandardized measured values from analyzers. Nevertheless, the ANN model showed overall higher performance than the ISTH criteria. Because of the small numbers of cases in set 2, it was inevitable that the 95% CI overlapped with the performance of ISTH criteria.

**Table 4:** Diagnostic performance of disseminated intravascular coagulation (DIC) diagnostic classifiers in this study.

Dataset	DIC criteria	DIC	Non-DIC	AUC (95% CI)		Diagnostic performance (95% CI)							
						Sensitivity (%)	Specificity (%)		PPV (%)		NPV (%)	Positive LR	Negative LR
Set 1 (n = 656)	ANN*			0.981 (0.973–0.989)		89.9 (85.3–93.5)	96.0 (93.7–97.7)		92.3 (88.3–95.1)		94.7 (92.4–96.3)	22.64 (14.18–36.14)	0.11 (0.07–0.15)
	Positive	205	17										
	Negative	23	411										
	ISTH			0.945 (0.929–0.962)		82.0 (76.4–86.8)	93.7 (91.0–95.8)		87.4 (82.7–90.9)		90.7 (88.1–92.8)	13.00 (8.98–18.82)	0.19 (0.15–0.25)
	Score ≥ 5	187	27										
	Score < 5	41	401										
	JMHW			0.943 (0.927–0.959)		91.2 (86.8–94.6)	84.4 (80.6–87.7)		75.6 (71.3–79.5)		94.8 (92.2–96.5)	5.83 (4.66–7.29)	0.10 (0.07–0.16)
	Score ≥ 7	208	67										
	Score < 7	20	361										
	JAAM			0.928 (0.909–0.946)		94.7 (91.0–97.3)	79.0 (74.8–82.7)		70.6 (66.6–74.3)		96.6 (94.2–98.0)	4.51 (3.74–5.43)	0.07 (0.04–0.12)
	Score ≥ 4	216	90										
	Score < 4	12	338										
Set 2 (n = 217)	ANN*				0.968 (0.945–0.986)	90.0 (81.2–95.6)		93.4 (87.9–97.0)		88.9 (80.9–93.8)	94.1 (89.2–96.9)	13.7 (7.25–25.87)	0.11 (0.06–0.22)
	Positive	72	9										
	Negative	8	128										
	ISTH				0.946 (0.916–0.976)	82.5 (72.4–90.1)		95.6 (90.7–98.4)		91.7 (83.3–96.0)	90.3 (85.3–93.8)	18.84 (8.56–41.46)	0.18 (0.11–0.29)
	Score ≥ 5	66	6										
	Score < 5	14	131										

ANN, artificial neural network; ISTH, the International Society on Thrombosis and Haemostasis; JMHW, the Japanese Ministry of Health and Welfare; JAAM, the Japanese Association for Acute Medicine; CI, Confidence Interval; AUC, area under curve; PPV, positive predictive value; NPV, negative predictive value; LR, likelihood ratio.

* Cut-off values for ANN model was determined at 0.501.

## Discussion

This study demonstrated ML-approach for DIC diagnosis to optimally integrate the DIC-related parameters. The established model enrolled 32 clinical parameters and the study shed light on the buried roles of overlooked clinical parameters in the scoring systems. However, the clinical implication of the enrolled variables remained uncertain to further investigation. We suggest that a number of additional cases with excluded variables should be obtained to precisely evaluate the role of anticoagulant use, bleeding, thrombosis, hematologic malignancy, immunologic insult, hepatic failure, obstetric complication, tissue damage, and vascular abnormalities in ML-approach. Therefore, the current ANN model may need to be further updated and validated with bigger data sets. Nevertheless, we believe that this approach may facilitate the diagnosis of DIC and the performance can be further improved by adding diverse training data and applying more advanced algorithms and parameters.

The major limitation of this study is DIC labeling procedure. Labeled results could be biased by the medical experts and by the limitation of retrospective approach. We employed supervised learning method which is generally used for classification and risk prediction in medicine [17]. In this approach, supervised labels determine the developed model. Although we labeled DIC status after expert agreements with careful medical record reviews, the labeled results can be incorrect or uncertain [34]. Moreover, we only enrolled the cases at the initial orders with varying elapsed time to diagnose, therefore consecutive monitoring of DIC profile was not possible except PLT changes. Because DIC is a rapid and dynamic change in blood vessels, an ML model reflecting consecutive changes of variable laboratory parameters would be developed in the future. These reasons may potentially play as limitations and could have affected the current model. Recently, several advancements in ML algorithms have been reported to overcome variations in human expert opinion. We expect that rapid advancement in ML algorithms may cover such issues in the future. ML cannot go beyond what’s contained in data. Meaning that more powerful and specific tests are still required for DIC diagnosis. Some studies have shown the usefulness of several methods in diagnosing DIC such as thromboelastography, clot waveform analysis, damage-associated molecular patterns, histone-DNA complexes, and circulating histones [13]. Additional data from such potential assays may also improve the performance.

Developing an AI system that gives contextual rationale is another important issue in the medical application. ANN is occasionally described as a ‘black box’ as it provides little explanatory insight into the variables [28]. However, recent studies illuminated substantial part of this ‘black box’ with a range of approaches. In order to provide intuitive information on clinical parameters, we calculated the relative importance and the values were mostly circumstantial to DIC features; absolute PLT count and changes, fibrin-related markers, PT prolongation were also important features in the ANN model, whereas fibrinogen level had relatively low importance. Additionally, some overlooked laboratory parameters such as PDW, RDW, and RUO parameters operated considerably in the ANN machinery. As a result of DIC progression, increases in PDW and MPV may be caused by a morphological transformation of PLT activation and young PLT production by megakaryopoiesis [35] that may explain the supportive role of PDW (3.28%) and MPV (1.48%). Additionally, mechanical damage to RBC during DIC progression such as schistocyte production may explain the importance of RDW (3.03%) [36]. Furthermore, TMA score (4.43%), an RUO parameter originally developed for the detection of TMA and reported to be linked to thrombocytopenia associated multiple organ failure, was revealed to be a supportive classifier for DIC [26]. DNI is another RUO parameter reflecting immature granulocyte percentages in circulating blood and has been reported to be a useful marker for sepsis. Because DIC commonly associated with sepsis, DNI (2.49%) may relate to this proportion in the ANN model [27]. Although most of the ranks of variable importance were understandable, it was difficult to interpret the relationships of some variables such as eosinophil (3.82%), lymphocyte (3.81%), and monocyte (3.31%) percentages. Those CBC differential count parameters may be negatively related to neutrophilia which is primarily induced by infection or malignancy and frequently accompanied with DIC.

Laboratory results vary among institutions even when the sample is identical. Because many laboratory parameters are required in this tool, standardization of their parameters remains problematic and must be addressed to reduce variation between institutions. Particularly, D-dimer assays, the salient variable in DIC evaluation, exploited various measuring principles with a lack of standardized calibrators and reporting units which lead to wide inter-laboratory and inter-method variability (Table A in S1 File) [37]. We believe that the best way is to use a normalized value such as scaled values or z-score, however, it is not practically possible for all laboratory parameters and is remained to be solved. This standardization issue should be always considered in the ML approach using laboratory parameters.

In conclusion, our study demonstrates a novel strategy to optimize the DIC diagnostic process with DIC-related parameters using ML-approach. The results showed some improvement of the diagnostic power in the retrospective design and provided additional insights into the importance of the DIC-related parameters. We believe this approach could be implemented in electrical health record system as a clinical decision support system in the near future. However, further prospective validation is required to assess the relationship between the ML-approach and their clinical benefit.

## Supporting information

S1 File Appendix A. Abbreviations, Text A. Description for clinical and laboratory variables used in this study, Table A. Information of the D-dimer assays used in this study, Text B. Online implementation of the model (http://optidic.net), Fig A. Online implementation of the ANN model for DIC evaluation.
(DOCX)

S2 File Datasets used in this study. (XLSX)
