# Supplementary material for: Machine learning-based diagnosis for disseminated intravascular coagulation (DIC): Development, external validation, and comparison to scoring systems
Source: PLoS One. 2018 May 2;13(5):e0195861. doi: 10.1371/journal.pone.0195861 (PMC5931474; doi:10.1371/journal.pone.0195861)

**Appendix A. Abbreviations**

DIC, disseminated intravascular coagulation; ML, machine learning; ANN, artificial neural network; AI, Artificial intelligence; ISTH, the International Society on Thrombosis and Haemostasis; JMHW, the Japanese Ministry of Health and Welfare; JAAM, the Japanese Association for Acute Medicine; CBC, complete blood count; RBC, red blood cell; MCV, mean corpuscular volume; MCH, mean corpuscular hemoglobin; MCHC, mean corpuscular hemoglobin concentration; RDW, red cell distribution width; WBC, white blood cell; PLT, platelet; MPV, mean platelet volume; PDW, platelet distribution width; PT, prothrombin time; INR, international normalized ratio; aPTT, activated partial thromboplastin time; FDP, fibrin degradation products; AT III, anti-thrombin III; RUO, research use only; LUC, large unstained cells; DNI, delta neutrophil index; TMA, thrombotic microangiopathy; APACHE, the Acute Physiology and Chronic Health Evaluation; SIRS, systemic inflammatory response syndrome; AUC, area under curve; CI, confidence interval;

**Text A. Description for clinical and laboratory variables used in this study**

Following physicians’ diagnostic procedure, all clinical variables were obtained at the same date with the DIC profile. Each case (development set, n=656; validation set, n=218) was reviewed for the below conditions and their binary status (0 or 1) was obtained based on the medical records. Furthermore, laboratory results were also obtained for the DIC profile parameters.

**1. DIC-related conditions: Binary data (yes or no)**

**Sepsis or infection**: Sepsis definition followed ‘the third international consensus for sepsis.’ Infection included any clinical evidence of infection from any microorganism

**Tissue damage**: physiologic damage in any organ including major trauma, fall, burn, laceration, ischemia, etc.

**Post-surgery**: major surgery, open brain or abdominal operations which could cause stressful condition

**Solid cancer**: diagnosis of cancer in solid organ with or without metastasis

**Hematologic malignancy**: acute or chronic leukemia, other plasma or lymphoproliferative neoplasm

**Liver failure**: diagnosis of acute liver failure, hepatic encephalopathy, or severe liver cirrhosis

**Obstetric complications**: pregnant women with pre-eclampsia or eclampsia, placental abruption

**Vascular abnormalities**: Aortic or cerebral aneurysm, ruptured aneurysm

**Immunologic insult:** Drug adverse event, severe auto-immune reaction, transplantation rejection, or severe transfusion reaction

References

1) Gando S, Levi M, Toh C-H. Disseminated intravascular coagulation. Nature Reviews Disease Primers 2016; 2:16037

2) Singer M, Deutschman CS, Seymour CW, Shankar-Hari M, Annane D, Bauer M, et al. The Third International Consensus Definitions for Sepsis and Septic Shock (Sepsis-3). JAMA 2016;315(8):801–810.

**2. Other clinical conditions: Binary data (yes or no)**

**Anti-coagulant use**: on anticoagulation with heparin, warfarin, or direct-oral anticoagulant (DOAC).

**Thrombosis**: all the cases categorized to ‘thrombosis’ confirmed by the evidence of US or CT scan findings at the evaluating point. This includes thrombosis event in any vessels such as pulmonary or cerebral embolism, and deep vein thrombosis.

**Bleeding:** both major and minor bleeding were included such as traumatic hemorrhage, brain hemorrhage, GI bleeding, post-partum hemorrhage and hemoptysis, etc

**Organ failure**: any clinical evidence in the medical records or lab findings for single or multiple organ failure including kidney, liver, lung, heart failure and encephalopathy. Unfortunately, SOFA score could not be obtained for all cases, therefore not evaluated.

**Systemic inflammatory response syndrome (SIRS)**: following the below SIRS criteria

(1) BT > 38℃ or < 36℃, (2) HR > 90 bpm, (3) RR > 20 or PaCO2 < 32 mmHg, (4) WBC > 12,000/mm³, < 4,000/mm³, or > 10% bands

**Intensive care unit (ICU) care**: the patients are currently in ICU unit.

**APACHE II score (if available)**: only calculated for the patient in ICU in our setting.

**3. Laboratory parameters: Numerical data [reference interval and used unit in the bracket]**

Reference intervals and result units vary among the institutions. Therefore, the predicted value produced by the ANN model may slightly differ without transforming the value to our setting. The reference intervals in the bracket are the values from development set.

**[Analyzer Information] These are the hematology analyzers collecting the data used in this study**

**Set 1: Severance Hospital (n = 656)**

**- CBC analyzer: ADVIA 2120i, Siemens Healthcare Diagnostics, IL, USA**

**- Global coagulation analyzer: ACL-TOP 750, Instrumentation Laboratory, MA, USA**

**Set 2: Gangnam Severance Hospital (n = 218)**

**- CBC analyzer: XN-9000, Sysmex, Kobe, Japan**

**- Global coagulation analyzer: CS-5100, Sysmex, Kobe, Japan**

For more detailed information, see manufacture’s instructor manual for each hematology analyzer.

1) Global coagulation test

**PT (Prothrombin Time):** PT % activity and INR were added in order to reduce inter-laboratory variability of PT values.

**PT (Sec)** [9.2 – 12.3 sec]: raw measured value in seconds, prolonged PT value was calculated through (patient PT – 12.3) sec

**PT % activity** [90 – 100%]: relative clotting activity compared to normal pooled plasma (NPP)

**INR** [0.91 – 1.16]: international normalized ratio

**APTT (Activated Partial Thromboplastin Time)** [26.8 – 40.6 sec]

**Thrombin time** [13.0 – 18.0 sec]

2) Fibrin related marker

**Fibrinogen**: measured by the Clauss method [200 – 400 mg/dL]

**D-dimer** [0-243 ng/mL]: D-dimer is measured in data display unit (DDU). Results measured in fibrinogen equivalent unit (FEU) can be transformed in DDU by dividing 1.75. (See TableS1)

D-dimer indicates more specific product of fibrin clots degradation. Usually, this parameter is used in (a) diagnosis of venous thromboembolism (VTE), (b) identification of individuals at increased risk of first thrombotic event (both arterial and venous), (c) identification of individuals at increased risk of recurrent VTE, (d) establishment of the optimal duration of secondary prophylaxis after a first episode of VTE, (e) pregnancy monitoring, and (f) diagnosis/monitoring of disseminated intravascular coagulation (DIC).

**FDP (Fibrin Degradation Product)** [0 – 5 mcg/mL]

**AT III (Anti-thrombin III)**: percent activity normalized by NPP [80 – 120 %]

3) Complete Blood Cell Count and related parameters

Red blood cell (RBC) indices

**RBC count** [4.4 – 6.1 × 10^6^/uL]

**Hemoglobin (Hb)** [13 – 17 g/dL]

**Hematocrit (Hct)** [40 – 52 %]

**Red cell distribution width (RDW)** [11.5 – 14.5 %]

Platelet (PLT) indices

**PLT count** [150 – 400 × 10^3^/uL]

**Platelet distribution width (PDW)** [25 – 65 %]: PDW is an index for platelet size variation. Currently, PDW values can be obtained by two different hematology analysers in fL (XN-3000; Sysmex, Japan) or % (ADVIA 2120i; Simens AG, Germany) unit. Although both units are not interchangeable, the correlation between two units could be presented as y (fL) = 0.18413 x (%) + 2.592; Pearson’s coefficient (r = 0.661).

**Mean platelet volume (MPV)** [7.4 – 12.1 fL]

**PLT changes**: % changes in PLT count [%/24hr], the values were calculated using the PLT count and their elapsed time between two samplings.

White blood cell differential counts (%): sum 100 %

**Neutrophil [39-74%], Lymphocyte [19-51%], Monocyte [3.3-10.8 %], Eosinophil [0-7%], Basophil [0-1.5%]**

4) Research use only (RUO) parameters

: RUO parameters are limitedly available in specific hematology analyzers only used for the purpose of research. We included these parameters in our model to maximize the diagnostic accuracy. There are three RUO parameters included in our model; TMA score, DNI, LUC.

1. **Thrombotic microangiopathy score (TMA score)**: this value ranges 0 to 4 in integer and automatically provided by specific analyzers according to the following criteria

(i) RDW > 14.5%, (ii) HDW > 3.2 g/dL, (iii) % microcytes (% micro) ≥ 0.4%, (iv) % hyperchromic red cells (% hyper) ≥ 1.9%

1. **Delta neutrophil index (DNI)**: this value reflects the immature granulocyte percentages in circulating blood

Immature granulocyte count: band neutrophil, metamyelocyte, myelocyte, promyelocyte

DNI = (Neutrophi % + Eosinophil %) - PMN cells % (in ADVIA2120i analyzer)

1. **Large unstained cells (%)**: this is the portion of large peroxidase-negative cells detected in the hematology analyzer. Sometimes, LUC includes large or reactive lymphocytes, monocytes or leukemic blasts

References

1) Buttarello M, Plebani M, Automated blood cell counts: state of the art. American Journal of Clinical Pathology. 2008;130(1):104-16.

2) Harris N, Jou JM, Devoto G, Lotz J, Pappas J, Wranovics D, et al. Performance evaluation of the ADVIA 2120 hematology analyzer: an international multicenter clinical trial. Laboratory Hematology. 2005;11(1):62-70.

3) Tripodi A. D-dimer testing in laboratory practice. Clinical Chemistry. 2011;57(9):1256-62

4) Seok Y, Choi JR, Kim J, Kim YK, Lee J, Song J, et al. Delta neutrophil index: a promising diagnostic and prognostic marker for sepsis. Shock 2012; 37:242-246.

5) Yoo J-H, Lee J, Roh KH, Kim HO, Song JW, Choi JR, et al. Rapid Identification of Thrombocytopenia-Associated Multiple Organ Failure Using Red Blood Cell Parameters and a Volume/Hemoglobin Concentration Cytogram. Yonsei Med J 2011; 52:845-850.

6) Thirup P. LUC, what is that? Large unstained cells. Clinical Chemistry. 1999;45(7):1100.

7) Lee E, Kim HS, Kang HJ, Kim M, Lee YK. Platelet Distribution Width from Two Automated Hematology Analysers: A Correlation Analysis. Journal of Laboratory Medicine and Quality Assurance 2017;39:42-46.

**Table A. Information of the D-dimer assays used in this study**

| Institution | Assay Name | Manufacturer | Methodology | Unit Type* | Manufacturer Cut-off | Reported Unit |
| --- | --- | --- | --- | --- | --- | --- |
| Set 1 | HemosIL  D-Dimer HS | Instrumentation Laboratory | Latex enhanced immunoturbidimetric immunoassay | DDU | < 243 | ng/mL |
| Set 2 | INNOVANCE  D-Dimer | Siemens AG | Quantitative, latex enhanced immunoturbidimetric immunoassay | FEU | < 500 | ng/mL |

* FEU, fibrinogen-equivalent units; DDU, data display unit

The methodologies currently used for D-dimer assessment are plagued with inter-laboratory and inter-method variability, as well as a lack of standardized calibrators and reporting units. The fibrinogen equivalent unit (FEU) is based on the mass of fibrinogen (340 kDa) and is approximately 1.75-fold higher than the D-dimer unit (DDU), which is based on the weight of the D-dimer. Because of these inconsistences, we calibrated D-dimer values in set 2 measured in FEU to DDU by dividing the factor 1.75 and used this transformed value for the model validation.

Reference

1) Riley RS, Gilbert AR, Dalton JB, Pas S, McPherson RA. Widely used types and clinical applications of D-dimer assay. Lab Med 2016; 47:90-102.

2) Hendriksen JM, Geersing GJ, van Voorthuizen SC, Oudega R, ten Cate-Hoek AJ, Joore MA, et al. The cost effectiveness of point-of-care D-dimer tests compared with a laboratory test to rule out deep venous thrombosis in primary care. Expert Rev Mol Diagn. 2015;15(1):125–136

**Text B. Online implementation of the model**

Our established model was published on a web page, called ‘OptiDIC’ (http://optidic.net), to provide supplementary information and research resources. Accessing this site, multiple cases can be tested by uploading a comma separated value (.csv) file on the provided template. Calculated values can be downloaded with a newly added column. Single cases can be tested by inputting patient data (Supplemental Fig 1A). For underlying conditions and clinical findings, the parameters have binary values. Laboratory results have appropriate input ranges and default units. Before a case is imputed, we highly recommend reading the manual (http://optidic.net/manual.php) to fully understand the use and application of this model. In particular, differences between hospitals’ patient composition, hematology analyzers or assay principles, inter-laboratory variations of results, report units, reference intervals, and missing values may affect the result, also optimal cut-off values may vary between institutions. Although the ANN model with RUO parameters showed better performance than the model without RUO parameters (AUC values 0.981 vs. 0.975), we offer two models: with and without RUO parameters, because RUO parameters are limited in specific analyzers. Users can choose one of the two ANN models for their dataset, but they should submit all variables for an appropriate evaluation. The PLT changes (%/24hrs) is automatically calculated using the previous PLT count (D-1), current PLT count, and elapsed time; this parameter can be unchecked if the previous PLT count is unavailable. On the results page, the calculated value (ranging from 0 to 1) with its interpretation is displayed. The closer to 1, the higher probability of DIC (Supplemental Fig 1B).

**Fig A.** Online implementation of the ANN model for DIC evaluation. (<http://optidic.net>)

(A) Input interface for required data fields (32 variables), (B) The result page for estimated value and its displayed interpretation.


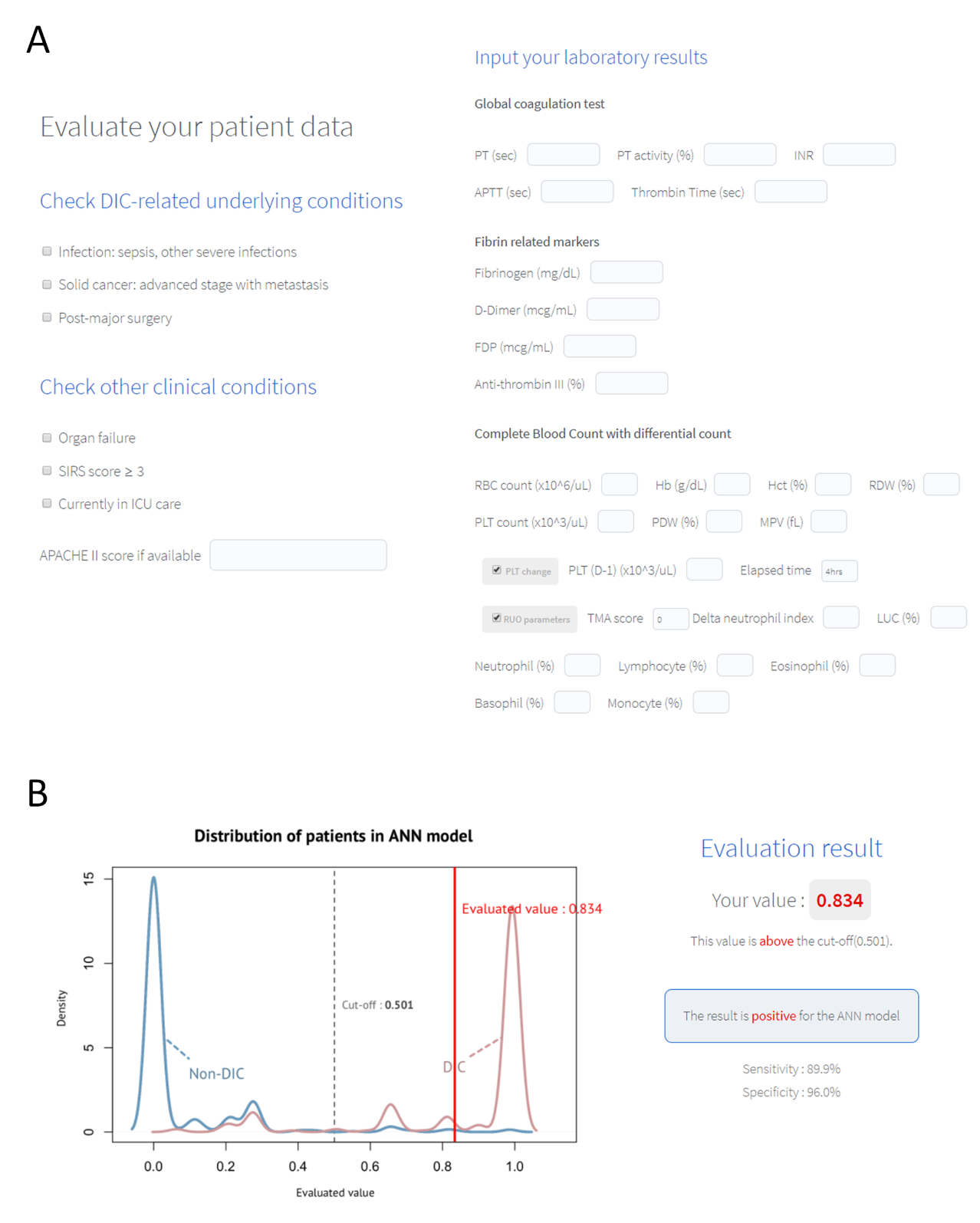

Supplement: S1 File — Abbreviations, Text A. Description for clinical and laboratory variables used in this study, Table A. Information of the D-dimer assays used in this study, Text B. Online implementation of the model (http://optidic.net), Fig A. Online implementation of the ANN model for DIC evaluation. (DOCX) [file pone.0195861.s001.docx]
